# Outcomes following Thulium vapoenucleation of large prostates

**DOI:** 10.1590/S1677-5538.IBJU.2015.0424

**Published:** 2016

**Authors:** Shane M. Pearce, Joseph J. Pariser, Rena D. Malik, Olufenwa J. Famakinwa, Doreen E. Chung

**Affiliations:** 1 Section of Urology, The University of Chicago Medical Center, Pritzker School of Medicine, Chicago, IL, USA; 2 Department of Urology, Columbia University Medical Center, New York, NY, USA

**Keywords:** Prostatic Hyperplasia, Thulium, Quality of Life, Treatment Outcome, Urinary Tract

## Abstract

**Introduction:**

Thulium laser VapoEnucleation of the prostate (ThuVEP) is an evolving surgical technique for BPH. Most studies have focused on outcomes in small to medium sized prostates and have originated from Europe and Asia. We sought to describe our experience with ThuVEP for very large prostates in a North American cohort.

**Materials and Methods:**

From December 2010 to October 2014, 25 men underwent ThuVEP using the CyberTM® (Quantastem, Italy) thulium laser, all with prostate volume >75mL. Data collected included patient demographics, comorbidities, intraoperative parameters, complications, and post-operative outcomes including maximum flow rate (Qmax), post-void residual (PVR), International Prostate Symptom Score (IPSS), and quality of life score (QoL) in one year of follow-up. Statistical analysis was done using Wilcoxon signed-rank test.

**Results:**

At baseline, mean age was 70±9 years and prostate size was 163±62g. Most patients (84%) were in retention and 10 (40%) patients were on anticoagulation. Seven (28%) patients went home the day of surgery (mean hospital stay: 1.2±1.2d). There were 2 intraoperative complications (8%), both cystotomies related to morcellation. Nine patients (36%) experienced a complication, all within 30 days. There were no Clavien ≥III complications. Significant improvements were seen in Qmax, PVR, IPSS, and QoL score at each time interval to 12-months following surgery (all p<0.05). Of 21 patients initially in retention, all were voiding at last follow-up.

**Conclusions:**

Our findings suggest that ThuVEP is an effective treatment for BPH in patients with large prostates with sustained results for one year.

## INTRODUCTION

Benign prostatic hyperplasia (BPH) and associated lower urinary tract symptoms (LUTS) are common problems, affecting 28% to 43% of men over age 60 and accounting for over $1 billion in health care costs (1). For men who fail medical management or experience sequelae of bladder outlet obstruction, the current gold standard surgical therapy remains transurethral resection of the prostate (TURP) for smaller prostates or open prostatectomy for very large organs (2). Traditional TURP is efficient and highly effective, however, it is associated with significant complications such as TUR syndrome, and blood transfusion rates remain significant at 2% to 8% in contemporary series (3, 4). Simple prostatectomy carries an even greater risk of perioperative morbidity and mortality (5). In 2009, McCullough et al. reported a 28% and 29% rate of post-operative hemorrhage in laparoscopic and open simple prostatectomy, respectively (6).

Modern laser therapy for BPH has advantages over TURP including decreased blood loss and minimal serum electrolyte changes resulting in fewer cardiovascular complications, decreased catheter time, shorter hospital stay and the ability to treat patients on anticoagulation (4, 7). Because of these potential advantages, there has been a shift in practice patterns with laser procedures accounting for 57% of surgical interventions for BPH, compared to traditional TURP which accounted for only 39% of interventions in 2005 (8).

Among laser therapies for prostate enucleation, holmium laser enucleation (HoLEP) has been studied most extensively, and found to provide similar clinical outcomes and decreased morbidity compared to simple prostatectomy for men with large prostates (9, 10). Widespread adoption of laser enucleation techniques has been hampered by a steep learning curve particularly in large prostates, which is supported by a recent multi-center trial identifying a steep learning curve for HoLEP exceeding 20 cases, with nearly half of participating centers choosing to abandon or not continue with the technique (11).

Use of the high-powered continuous-wave thulium laser for treatment of BPH was first described in 2005, followed by multiple case series describing the use of the laser for both prostate vaporization (ThuVP) and enucleation (ThuVEP) procedures. ThuVEP has produced favorable clinical outcomes with minimal side effects in several studies from Europe and Asia (12–14). Rausch et al. recently reported improvements in International Prostate Symptom Score (IPSS), quality of life (QOL), post void residual (PVR) and maximum urine flow at 24 months in a series of 234 patients who underwent ThuVEP with a mean prostate size of 85mL (15). Interestingly, they found that small prostate size (<80mL) was a predictor of complications and treatment failure.

Initial experiences with ThuVEP have been positive, but highlighted the question of patient selection. Laser vaporization procedures appear to be supplanting TURP for treatment of smaller prostates. However, few studies have examined the outcomes of ThuVEP in men with larger prostates, when the alternative treatment would be simple prostatectomy. This study was performed to assess the feasibility, safety, and efficacy of ThuVEP in men with large prostates at two teaching hospitals in the United States.

## MATERIALS AND METHODS

Institutional review board approval was obtained, and the medical records of 25 men who underwent ThuVEP from December 2010 to October 2014 by a single surgeon at two teaching hospitals were retrospectively analyzed. The surgeon had extensive previous experience with TURP, laser vaporization of the prostate, and HoLEP techniques, however, this was the surgeon's initial experience with the ThuVEP technique. All patients had an estimated prostate volume >75mL. Indications for surgery were history of urinary retention (23 or 92%) and LUTS unresponsive to medical management (2 or 8%). Baseline data were collected on demographics, medications, and comorbidities including American Society of Anesthesiologist Physical Status classification (ASA) and age-adjusted Charlson Comorbidity Index (CCI).

Preoperative urologic evaluation included a complete history, physical exam, digital rectal exam (DRE), IPSS, IPSS-QoL Index, PVR, uroflowmetry, serum prostate-specific antigen (PSA), urine culture, transrectal ultrasound and cystoscopy. Urodynamics was performed in select patients with comorbidities or incontinence, according to International Continence Society Guidelines (16–18). In appropriately aged patients at risk of clinically significant prostate cancer, based on a concerning DRE or elevated PSA, a 14-core transrectal ultrasound guided biopsy of the prostate was performed preoperatively to exclude malignancy. Anti-platelet agents such as high-dose aspirin and clopidogrel were either held or continued based on cardiovascular risk assessment by internal medicine specialists. Patients taking warfarin were bridged to enoxaparin or heparin during the perioperative period.

ThuVEP was performed using a 26F continuous flow cystoscopy with a laser bridge. We used the CyberTM® laser (Quanta System, Italy) with an 800 or 1000μm laser fiber. Vapoenucleation was performed in a systematic fashion. All patients were under general or spinal anesthesia in the dorsal lithotomy position. Following identification of the ureteral orifices, with the laser set at 80W, incisions were made at the 5 and 7 o'clock positions starting at the bladder neck and extending to the level of the verumontanum. The incisions were connected distally and the median lobe was then enucleated at the level of the surgical capsule of the prostate from distal to proximal. The distal extent of the lateral lobe to be enucleated was then marked. Incisions were made at the 2 and 10 o'clock positions from bladder neck connecting to the previously made distal markings. Similar to the median lobe enucleation, the lateral lobes were then enucleated from distal to proximal. Anterior tissue, between the 10 and 2 o'clock position, and any irregularity in the prostate bed was then vaporized with laser set to 120W down to the level of the surgical capsule. Any excess tissue at the apex near the verumontanum was vaporized on a setting of 80W. Meticulous hemostasis was obtained with a laser power of 40W to 60W. Tissue morcellation was then performed through a 26F nephroscope using the Piranha® morcellation system (Wolf). A 20F two-way Foley catheter was inserted and manually irrigated to confirm adequate hemostasis. Continuous bladder irrigation was routinely performed in our early experience (n=8), but was later deemed to be unnecessary. Patients were typically discharged on the day of surgery or on post-operative day one depending on comorbidities, patient wishes, degree of hematuria, and access to care. The catheter was typically removed at the first post-operative clinic appointment.

Perioperative parameters included operative time, laser time (exact amount of time laser was active), laser energy, enucleation specimen weight, post-operative serum hemoglobin and serum sodium, length of stay and catheterization time. Post-operative functional outcomes were assessed at 1 month, 3 months, 6 months and 12 months including IPSS, QoL, Qmax and PVR. PSA was performed at baseline and at post-operative months 3 to 12. All complications were classified according to the Clavien-Dindo grading system (19). All statistical analysis was performed using Stata®, version 13 (Statacorp, College Station, TX). The Wilcoxon signed-rank test was used to analyze changes in outcome measures between time points, and a p-value of <0.05 was considered statistically significant.

## RESULTS

Twenty-five patients underwent ThuVEP and were included in analysis. Table-1 describes the baseline characteristics of the study participants. The mean age was 70±9 years and mean BMI was 28±6kg/m^2^. Mean ASA was 2.6±0.6 and age-adjusted CCI was 4.2±2.0, with coronary artery disease and diabetes mellitus present in 20% (n=5) and 32% (n=8) of patients, respectively. Ten patients (40%) were on anticoagulation peri-operatively including aspirin alone (n=7), aspirin plus clopidogrel (n=2), and warfarin bridged to enoxaparin (n=1).

**Table 1:** Baseline Characteristics.

Parameter (n=25)	Mean±SD (range)
Age	70±9 (53-90)
BMI (kg/m2)	28±6 (18-42)
CCI (age adjusted)	4.2±2.0 (2-11)
ASA	2.6±0.6 (1-4)
Urinary retention (N [%])	23 (92)
On anticoagulation (N [%])	10 (40)
IPSS	19.3±7.3 (8-30)
QoL	5.2±1.3 (2-6)
Qmax (mL/sec)	4.2±4.2 (1-18)
PVR (mL)	355±274 (21-1000)
PSA (ng/mL)	7.4±4.7 (0.6-18)
Prostate Volume (mL)	163±62 (77-327)
Bladder Outlet Obstructive Index	87±48 (11-220)

**BMI** = body mass index; **IPSS** = international prostate symptom score; **QoL** = quality of life; **Qmax** = maximum flow rate; **PVR** = post-void residual; **PSA** = prostate-specific antigen; **CCI** = age-adjusted Charlson Comorbidity Index

The mean prostate volume was 163±62mL and mean PSA was 7.4±4.7ng/mL. Baseline IPSS was 19.3±7.3, QoL was 5.2±1.3, Qmax was 4.2±4.2mL/sec and PVR was 355±274mL. Four patients (16%) had a history of previous bladder outlet procedure and all were in retention preoperatively, having failed previous voiding trials. Eighteen (72%) were taking 5α-reductase inhibitors preoperatively and 23 (92%) were taking an α-blocker prior to surgery. Urodynamics was performed in twenty patients (80%) and the mean Bladder Outlet Obstructive Index was 87±48. Among patients who underwent urodynamics, thirteen (65%) were diagnosed with detrusor overactivity.

Operative outcomes and perioperative data are summarized in Table-2. The mean operative time was 204±58 minutes with mean 63±20 minutes of laser time. The total mean laser energy used was 347±123 kilojoules. The mean enucleated tissue weight was 47±29g with a mean morcellation time of 25±17 minutes. One patient (4%) was found to have incidental prostate cancer (Gleason score 3+3) on final pathology and the remaining 24 (96%) had benign tissue. Postoperative serum sodium concentration did not change significantly from baseline (p=0.7), but there was a decrease in serum hemoglobin (-0.6±1.1, p=0.01). Eight patients (32%) were placed on continuous bladder irrigation as per routine early in our experience, but this practice was eventually felt to be unnecessary. The majority of patients were discharged on the day of surgery (n=7, 28%) or on postoperative day one (n=13, 52%), and the mean length of stay was 1.2±1.2 days. The catheter was typically removed at the first postoperative follow-up appointment with a mean catheter time of 6.5±2.7 days. The mean postoperative PSA was 2.9±2.3ng/mL at a mean of 6.8±3.6 months after surgery, which was significantly decreased from baseline (7.4±4.7ng/mL, p<0.01).

**Table 2:** Operative and perioperative Outcomes.

Outcome	Mean±SD (range)
Operative time (min)	204±58 (124-332)
Laser time (min)	63±20 (28-104)
Laser energy (kJ)	347±123 (163-639)
Morcellation time (min)	25±17 (8-60)
Enucleation Weight (g)	47±29 (10-130)
Change in serum sodium (mM)	0.0±3.1 (-9-5)
Change in serum hemoglobin (g/dL)*	-0.6±1.1 (-2.8-1.5)
Hospital stay (days)	1.2±1.2 (0-5)
Catheter time (days)	6.5±2.7 (3-16)
Mean postoperative PSA (ng/mL)*	2.9±2.3 (0.7-8.2)

* p<0.05 postoperative hemoglobin and PSA compared to baseline

Functional outcome measures including objective voiding parameters and subjective patient reported outcomes are shown in Table-3. With respect to subjective measures, significant improvements from baseline were observed for IPSS and QoL at all time points (p<0.05). Similarly, Qmax and PVR demonstrated improvements from baseline at one-, three-, six-, and twelve-month follow-up visits (p<0.05). All 20 patients initially in retention were voiding at last follow-up.

**Table 3:** Functional outcome measures.

Outcome (mean±SD [range])	Baseline	1 month*	3 months*	6 months*	12 months*
IPSS	19.3±7.3 (8-30)	6.5±4.4 (1-16)	5.8±4.1 (0-12)	5.4±5.6 (1-20)	47±4.6 (0-13)
QoL	5.2±1.3 (2-6)	1.7±1.4 (0-5)	2.3±2.0 (0-7)	1.2±1.0 (0-3)	1.2±1.3 (0-4)
Qmax (mL/sec)	4.2±4.2 (1-18)	14.6±8.5 (3-32)	17.7±7.2 (5-31)	15.0±9.9 (4-32)	20.1±10.4 (3-37)
PVR (mL)	355±274 (21-1000)	107±169 (0-737)	67±93 (0-284)	102±175 (0-581)	50±58 (0-159)

* p<0.05 for all outcomes at each time point compared to baseline

We observed 2 intraoperative complications (8%) are described in Table-4, which were both cystotomies that occurred due to inadvertent engagement with the bladder wall during morcellation. Both were identified intraoperatively, managed successfully with catheter drainage alone and resulted in no further adverse sequelae. A total of 9 patients experienced 30-day complications (36%) including 10 complications overall. There were 9 Clavien grade I complications, 1 grade II complication and no grade ≥III complications. The grade I complications included 5 culture-proven UTIs (20%), all of which resolved with oral antibiotic treatment, 3 patients failed initial voiding trial (12%) requiring re-catheterization and 1 patient (4%) experienced gross hematuria with clot retention requiring bladder irrigation. The grade II complication was a single blood transfusion for a patient who had significant cardiac comorbidity and was anemic preoperatively with serum hemoglobin of 9.6g/dL. Postoperative serum hemoglobin was 10.0g/dL. He became tachypneic and tachycardic in the recovery room and was transfused based on cardiology and anesthesiology recommendations. No complications were observed after 30-days.

**Table 4:** Complications.

Complication	Frequency (%)
Intraoperative	2 (8)
Any 30-d complication	9 (36)
UTI	5 (20)
Urinary retention requiring re-catheterization	3 (12)
Clot retention	1 (4)
Transfusion	1 (4)
Late complication (30d-12mo)	0 (0)

## DISCUSSION

Laser enucleation techniques such as ThuVEP and HoLEP have emerged over the last 10 years as viable treatment options for BOO based on evidence from prospective clinical trials. HoLEP has been more extensively studied and is considered to be a comparable alternative to simple prostatectomy in patients with large prostates and to TURP in patients with prostates <75mL (9, 10). The thulium laser is a relatively new technology with several potential advantages over alternative lasers for the treatment of BPH such as favorable hemostatic properties, a relatively shallow depth of thermal damage (20) and the ability to perform hybrid procedures utilizing both vaporization and resection properties of the laser (21, 22). Bach et al. first described the ThuVEP technique in 2009 as a safe and durable procedure (23), and since then multiple case series from Europe and Asia have reported outcomes, with the majority of publications coming from a few centers (13, 15, 21, 24–29).

In our patient cohort with a very large mean prostate size (163mL), significant comorbid disease (mean CCI 4.2 and ASA 2.6) and clinical evidence of BOO (84% in retention), ThuVEP offers improvement in voiding parameters with a favorable morbidity profile. To our knowledge, this is the first study to evaluate the safety, feasibility, and efficacy of ThuVEP in a North American patient population, supporting the technique as a possible alternative to TURP and simple prostatectomy for the surgical management of BPH. Our findings also support several potential advantages of ThuVEP over these traditional approaches including fewer electrolyte changes, less blood loss, and shorter length of stay.

We demonstrated an improvement in objective and subjective voiding parameters out to 1 year of follow-up. The observed improvement in obstructive voiding after ThuVEP is comparable to HoLEP (30), Greenlight laser vaporization (31), TURP (32) and simple prostatectomy (33). At 12 months follow-up, IPSS and QoL scores improved by 14.6 and 4.0 points, respectively. Objective voiding parameters also improved, with a 15.9mL/sec increase in mean Qmax and a 305mL reduction in PVR. Our reported improvement in postoperative voiding parameters is similar to results in other ThuVEP series, supporting the effectiveness of the procedure (25). Additionally, no patients in our series required reoperation, which is consistent with the 0% to 2.4% overall revision rates reported in other ThuVEP series with at least 1 to 2 years of follow-up (25). The 61% reduction in postoperative PSA from preoperative baseline PSA is similar to reductions seen in other early ThuVEP experiences, providing further evidence for the efficacy of the procedure (23, 26). While there was a discrepancy between the mean prostate volume (163mL) and the enucleation weight (46g) in our series, this was likely related the large amount of tissue vaporized during ThuVEP.

In regard to safety, we reported an intra-operative complication rate of 8% and a 30-day complication rate of 36%, which is comparable to the rate (overall complication rate of 30.9%) reported by Gross et al. in a large (n=1080), prospective evaluation of complications after ThuVEP using an operative technique similar to the present study (29). When analyzing the severity of complications, the vast majority of complications were Clavien grade I. UTI (20%) and urinary retention after initial decatheterization (12%) represented the most common complications, similar to previous reports by Gross et al. (29). Interestingly, they reported a 41.7% overall complication rate during their first 216 cases, which improved to 19.4% during the last 216 cases. They also demonstrated that transfusion rates and urinary retention decreased over time, but the rate of postoperative UTI remained stable. Our initial morbidity profile compares favorably to this large study, particularly when taken in the context of a well demonstrated learning curve for ThuVEP (27) and the similar HoLEP technique (34, 35).

In our series, there was a statistically significant drop in serum hemoglobin from baseline (12.6g/dL) to postoperative (12.0g/dL), however, this did not seem to be clinically significant as only one patient (who may have required transfusion even preoperatively) received a postoperative blood transfusion. Additionally, this patient actually had a small increase in hemoglobin from baseline to immediately following surgery. The 0.6g/dL decrease in serum hemoglobin is similar to the change seen by Gross et al. after ThuVEP (1.1g/dL) and multiple series after Greenlight laser vaporization (0.7-1.2g/dL) (29, 31, 36). Despite ongoing oral anticoagulation in 40% of our patients, blood loss was minimal, transfusion rate was appropriately low and few intraoperative complications were observed. Additionally, the serum sodium concentration in our study did not change significantly from baseline to after surgery, suggesting that ThuVEP is not generally associated with major electrolyte disturbances.

Our study is unique in that 28% of our patients were treated as outpatient, and 52% stayed in the hospital just for one night, which resulted in a mean length of stay and catheterization time of 1 day and 7 days, respectively. While our hospital stay is significantly shorter and catheterization time is somewhat longer than other HoLEP and ThuVEP series (29, 30), this likely represents geographic variation in practice patterns in the United States compared to China and Europe. For example, in the German health system, reimbursement is by case-based lump sums, requiring a minimum of two overnight hospital stays for full reimbursement. Additionally, many European studies describe routinely keeping the patient in the hospital until the catheter is removed and the patient has voided normally. Our preferred practice, particularly later in the experience, was to discharge patients home on the day of surgery and remove the catheter at the first postoperative follow-up appointment.

Our early experience suggested ThuVEP was safe and effective in men with large prostates (mean prostate volume 163mL), even in the setting of ongoing oral anticoagulation. The mean prostate size in the present series is significantly greater than other large ThuVEP series (51-110mL) (15, 29, 37). We confirmed that ThuVEP appears to be a size independent procedure with significant improvements in all voiding parameters and minimal perioperative morbidity in a patient cohort with the largest mean preoperative prostate size in the literature. Rausch et al. recently examined prostate size as a predictor of adverse surgical outcomes after ThuVEP in a total of 234 patients with a mean preoperative prostate size of 85mL (15). They found that prostate size <80mL was associated with treatment failure, and prostate size <50mL independently predicted complications. While we cannot comment on risk factors for adverse outcomes because of a relatively small sample size, no patients experienced treatment failure (defined as reoperation or placement of indwelling catheter) and the thirty-day complication rate for patients with a prostate volume ≥149mL (median in our series) was 31%, which is comparable to the rate in the entire cohort (36%). A recent large multicenter study evaluated the outcomes of photoselective vaporization of the prostate (PVP) using the 180W-XPS system in men with prostate volume ≥80mL (n=387) compared to prostate volume <80mL (n=739) (38). They concluded that the XPS system was safe and effective for men with prostate size ≥80mL (median 108mL) producing similar improvements in symptoms and retreatment rates at 2 years follow-up compared to men with prostate volume <80mL. However, men with larger prostates were at a higher risk of intra-operative conversion to TURP likely due to obscured vision from bleeding. The mean prostate volume in our study was notably larger than this study and no patients in our series required conversion to traditional TURP.

Taken as a whole, our results suggest that ThuVEP is safe and effective in highly comorbid patients, including many who were on anticoagulation peri-operatively and in urinary retention at baseline. The procedure was effective in men with very large prostates and produced improvement in patient reported outcomes and objective voiding parameters with 1 year of follow-up. We demonstrate that ThuVEP can be performed in a North American patient population on an outpatient basis.

Limitations of our study include its retrospective nature, relatively small sample size, lack of a comparison group and short follow-up. As a single surgeon series, our results may not be applicable to all settings. Larger comparative studies are needed.

## CONCLUSION

Thulium enucleation of the prostate is effective and yields improvement in patient reported outcomes and objective voiding parameters out to 1 year for the treatment of BPH in men with very large prostates.
